# Ten Simple Rules for Selecting a Postdoctoral Position

**DOI:** 10.1371/journal.pcbi.0020121

**Published:** 2006-11-24

**Authors:** Philip E Bourne, Iddo Friedberg

You are a PhD candidate and your thesis defense is already in sight. You have decided you would like to continue with a postdoctoral position rather than moving into industry as the next step in your career (that decision should be the subject of another “Ten Simple Rules”). Further, you already have ideas for the type of research you wish to pursue and perhaps some ideas for specific projects. Here are ten simple rules to help you make the best decisions on a research project and the laboratory in which to carry it out.

## Rule 1: Select a Position that Excites You

If you find the position boring, you will not do your best work—believe us, the salary will not be what motivates you, it will be the science. Discuss the position fully with your proposed mentor, review the literature on the proposed project, and discuss it with others to get a balanced view. Try and evaluate what will be published during the process of your research. Being scooped during a postdoc can be a big setback. Just because the mentor is excited about the project does not mean you that will be six months into it.

## Rule 2: Select a Laboratory That Suits Your Work and Lifestyle

If at all possible, visit the laboratory before making a decision. Laboratories vary widely in scope and size. Think about how you like to work—as part of a team, individually, with little supervision, with significant supervision (remembering that this is part of your training where you are supposed to be becoming independent), etc. Talk to other graduate students and postdoctoral fellows in the laboratory and determine the work style of the laboratory. Also, your best work is going to be done when you are happiest with the rest of your life. Does the location of the laboratory and the surrounding environment satisfy your nonwork interests?

## Rule 3: Select a Laboratory and a Project That Develop New Skills

Maximizing your versatility increases your marketability. Balance this against the need to ultimately be recognized for a particular set of contributions. Avoid strictly continuing the work you did in graduate school. A postdoctoral position is an extension of your graduate training; maximize your gain in knowledge and experience. Think very carefully before extending your graduate work into a postdoc in the same laboratory where you are now—to some professionals this raises a red flag when they look at your resume. Almost never does it maximize your gain of knowledge and experience, but that can be offset by rapid and important publications.

## Rule 4: Have a Backup Plan

Do not be afraid to take risks, although keep in mind that pursuing a risky project does not mean it should be unrealistic: carefully research and plan your project. Even then, the most researched, well-thought-out, and well-planned project may fizzle; research is like that. Then what? Do you have a backup plan? Consider working on at least two projects. One to which you devote most of your time and energy and the second as a fallback. The second project should be more of the “bread and butter” type, guaranteed to generate good (if not exciting) results no matter what happens. This contradicts *Rule 1,* but that is allowed for a backup plan. For as we see in *Rule 5,* you need tangible outcomes.

## Rule 5: Choose a Project with Tangible Outcomes That Match Your Career Goals

For a future in academia, the most tangible outcomes are publications, followed by more publications. Does the laboratory you are entering have a track record in producing high-quality publications? Is your future mentor well-respected and recognized by the community? Talk to postdocs who have left the laboratory and find out. If the mentor is young, does s/he have the promise of providing those outcomes? Strive to have at least one quality publication per year.

## Rule 6: Negotiate First Authorship before You Start

The average number of authors on a paper has continued to rise over the years: a sign that science continues to become more collaborative. This is good for science, but how does it impact your career prospects? Think of it this way. If you are not the first author on a paper, your contribution is viewed as 1/*n* where *n* is the number of authors. Journals such as this one try to document each author's contributions; this is a relatively new concept, and few people pay any attention to it. Have an understanding with your mentor on your likelihood of first authorship before you start a project. It is best to tackle this problem early during the interview process and to achieve an understanding; this prevents conflicts and disappointments later on. Don't be shy about speaking frankly on this issue. This is particularly important when you are joining an ongoing study.

## Rule 7: The Time in a Postdoctoral Fellowship Should Be Finite

Mentors favor postdocs second only to students. Why? Postdocs are second only to students in providing a talented labor pool for the least possible cost. If you are good, your mentor may want you to postdoc for a long period. Three years in any postdoc is probably enough. Three years often corresponds to the length of a grant that pays the postdoctoral fellowship, so the grant may define the duration. Definitely find out about the source and duration of funding before accepting a position. Be very wary about accepting one-year appointments. Be aware that the length of a postdoc will likely be governed by the prevailing job market. When the job market is good, assistant professorships and suitable positions in industry will mean you can transition early to the next stage of your career. Since the job market even a year out is unpredictable, having at least the option of a three-year postdoc fellowship is desirable.

## Rule 8: Evaluate the Growth Path

Many independent researchers continue the research they started during their postdoc well into their first years as assistant professors, and they may continue the same line of work in industry, too. When researching the field you are about to enter, consider how much has been done already, how much you can contribute in your postdoc, and whether you could take it with you after your postdoc. This should be discussed with your mentor as part of an ongoing open dialog, since in the future you may be competing against your mentor. A good mentor will understand, as should you, that your horizon is independence—your own future lab, as a group leader, etc.

## Rule 9: Strive to Get Your Own Money

The ease of getting a postdoc is correlated with the amount of independent research monies available. When grants are hard to get, so are postdocs. Entering a position with your own financing gives you a level of independence and an important extra line on your resume. This requires forward thinking, since most sources of funding come from a joint application with the person who will mentor you as a postdoc. Few graduate students think about applying for postdoctoral fellowships in a timely way. Even if you do not apply for funding early, it remains an attractive option, even after your postdoc has started with a different funding source. Choosing one to two potential mentors and writing a grant at least a year before you will graduate is recommended.

## Rule 10: Learn to Recognize Opportunities

New areas of science emerge and become hot very quickly. Getting involved in an area early on has advantages, since you will be more easily recognized. Consider a laboratory and mentor that have a track record in pioneering new areas or at least the promise to do so.

