# Correction: Ten Simple Rules for Selecting a Postdoctoral Position

**DOI:** 10.1371/journal.pcbi.0020181

**Published:** 2006-12-29

**Authors:** Philip E Bourne, Iddo Friedberg

In *PLoS Computational Biology,* volume 2, issue 11: doi: 10.1371/journal.pcbi.0020121

In the author affiliation section, Dr. Iddo Friedberg should have been listed as a research scientist (instead of as a research assistant).

In the section called **Rule 1: Select a Position That Excites You**, the sentence, “Just because the mentor is excited about the project does not mean that you will be six months into it.”, had the two words “that” and “you” switched.

